# *Ureaplasma diversum* Genome Provides New Insights about the Interaction of the Surface Molecules of This Bacterium with the Host

**DOI:** 10.1371/journal.pone.0161926

**Published:** 2016-09-07

**Authors:** Lucas M. Marques, Izadora S. Rezende, Maysa S. Barbosa, Ana M. S. Guimarães, Hellen B. Martins, Guilherme B. Campos, Naíla C. do Nascimento, Andrea P. dos Santos, Aline T. Amorim, Verena M. Santos, Sávio T. Farias, Fernanda Â. C. Barrence, Lauro M. de Souza, Melissa Buzinhani, Victor E. Arana-Chavez, Maria E. Zenteno, Gustavo P. Amarante-Mendes, Joanne B. Messick, Jorge Timenetsky

**Affiliations:** 1 Department of Microbiology, Institute of Biomedical Science, University of São Paulo, São Paulo, Brazil; 2 Multidisciplinary Institute of Health, Universidade Federal da Bahia, Vitória da Conquista, Brazil; 3 University of Santa Cruz (UESC), Campus Soane Nazaré de Andrade, lhéus, Brazil; 4 Department of Animal Health and Preventive Veterinary Medicine, College of Veterinary Medicine and Animal Science, University of São Paulo, São Paulo, Brazil; 5 Department of Comparative Pathobiology, Purdue University, West Lafayette, Indiana, United States of America; 6 Evolutionary Genetics Laboratory, Department of Molecular Biology, Federal University of Paraíba, João Pessoa, Paraíba, Brazil; 7 Laboratory of Biology of Mineralized Tissues, Institute of Biomedical Sciences, University of São Paulo, São Paulo, Brazil; 8 Instituto de Pesquisa Pelé Pequeno Príncipe—Faculdades Pequeno Príncipe, Curitiba, Brazil; 9 Department of Dental Materials, School of Dentistry, University of São Paulo, São Paulo, Brazil; 10 Laboratory of Cellular and Molecular Biology, Institute of Biomedical Science, University of São Paulo, São Paulo, Brazil; University of North Dakota School of Medicine and Health Sciences, UNITED STATES

## Abstract

Whole genome sequencing and analyses of *Ureaplasma diversum* ATCC 49782 was undertaken as a step towards understanding *U*. *diversum* biology and pathogenicity. The complete genome showed 973,501 bp in a single circular chromosome, with 28.2% of G+C content. A total of 782 coding DNA sequences (CDSs), and 6 rRNA and 32 tRNA genes were predicted and annotated. The metabolic pathways are identical to other human ureaplasmas, including the production of ATP via hydrolysis of the urea. Genes related to pathogenicity, such as urease, phospholipase, hemolysin, and a Mycoplasma Ig binding protein (MIB)—Mycoplasma Ig protease (MIP) system were identified. More interestingly, a large number of genes (n = 40) encoding surface molecules were annotated in the genome (lipoproteins, multiple-banded antigen like protein, membrane nuclease lipoprotein and variable surface antigens lipoprotein). In addition, a gene encoding glycosyltransferase was also found. This enzyme has been associated with the production of capsule in mycoplasmas and ureaplasma. We then sought to detect the presence of a capsule in this organism. A polysaccharide capsule from 11 to 17 nm of *U*. *diversum* was observed trough electron microscopy and using specific dyes. This structure contained arabinose, xylose, mannose, galactose and glucose. In order to understand the inflammatory response against these surface molecules, we evaluated the response of murine macrophages J774 against viable and non-viable *U*. *diversum*. As with viable bacteria, non-viable bacteria were capable of promoting a significant inflammatory response by activation of Toll like receptor 2 (TLR2), indicating that surface molecules are important for the activation of inflammatory response. Furthermore, a cascade of genes related to the inflammasome pathway of macrophages was also up-regulated during infection with viable organisms when compared to non-infected cells. In conclusion, *U*. *diversum* has a typical ureaplasma genome and metabolism, and its surface molecules, including the identified capsular material, represent major components of the organism immunopathogenesis.

## Introduction

*Ureaplasma diversum* is a bovine ureaplasma that was first isolated in 1969. Initially, it was defined as a non-pathogenic species, but recently it has been shown to cause damage to bovine tissue cells and organs [1–9]. *U*. *diversum* is frequently found in the genital tract of cattle and is associated with major genital disorders in these animals [5, 10, 11]. Cows infected with *U*. *diversum* have shown infertility, placentitis, fetal alveolitis, and abortion or birth of weak calves [7, 12, 13]. In bulls, *U*. *diversum* may cause low sperm motility, seminal vesiculitis, and epididymitis [4, 6, 9, 13]. However, despite the description of these possible causal associations, the relationship of *U*. *diversum* and reproductive disorders in bovine remains controversial, mainly because high rates of positive vaginal cultures were also detected in animals with normal reproductive rates [14].

*U*. *diversum* is a facultative intracellular microbe, i.e. can be detected inside cells or adhered to their surfaces [15]. Recently, we have shown that the invasion of HEp-2 cells by this organism may lead to apoptosis [1], but as this phenomenon varied overtime. Thus, it is believed that *U*. *diversum* exerts a temporal modulation of the host programmed cell death. Invasion of bovine spermatozoids by *U*. *diversum* has also been linked to low sperm viability, suggesting that *U*. *dive*rsum may contribute to the death of these cells [4]. *U*. *diversum* also was capable of inducing significant TNF-alpha production in the uterus of experimentally infected mice [16], which indicates that the presence of this microorganism in the reproductive tract of females may significantly alter the homeostasis of the uterus microenvironment. Nevertheless, the molecular mechanisms by which this organism exerts its virulence and pathogenicity on such cells and tissues are mostly unknown [1, 15, 17, 18]. Very little genetic information of this bacterium is currently available [19, 20]. Therefore, the whole genome sequencing of *U*. *diversum* was undertaken as the first step towards understanding the mechanisms by which this microorganism causes disease and establishes infection, as well as to gain new insights into the biochemical pathways.

## Results and Discussion

### General genome features

**Fig 1:**
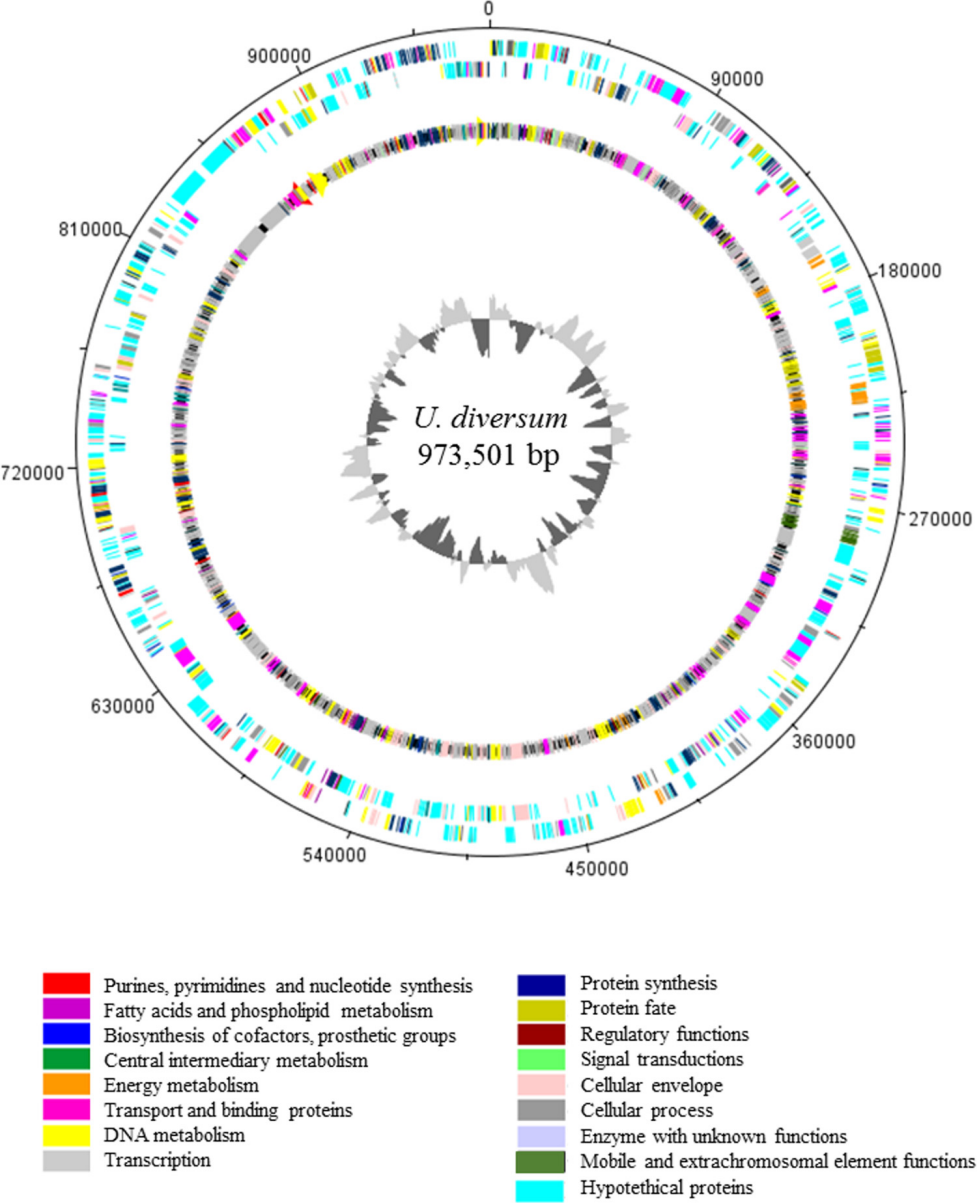
Diagram of the overall structure of *Ureaplasma diversum* ATCC 49782 genome. The *dnaA* gene is at position zero. The distribution of genes is depicted on two outermost concentric circles. First concentric circle: predicted coding DNA sequences (CDSs) on the plus strand. Second concentric circle: predicted CDSs on the minus strand. The innermost circle represents the GC skew. The figure was generated using DNAPlotter version 1.4 from Artemis 12.0, Sanger Institute.

The general genome features of *U*. *diversum* ATCC 49782 are summarized in Table 1 and Fig 1. The complete genome contains 973,501 bp in a single circular chromosome, with a low G+C content of 28.2%. It uses the opal stop codon (UGA) for tryptophan. A total of 782 coding DNA sequences (CDS), and 6 rRNA and 32 tRNA genes were predicted and annotated. Four hundred and seventy CDSs (60.1%) have putative functions, while 272 CDSs (35.7%) encode for hypothetical proteins. Predicted CDSs are summarized by role in Table 2.

**Table 1:** General features of the genome of *Ureaplasma diversum* ATCC 49782 compared to human ureaplasmas and other members of *Mycoplasma*, *Acholeplasma* and *Phytoplasma* species.

Parameter	Udv	Upa	Uua	Mmy	Mbo	Mpn	Mge	Mho	Mgal	Msy	Mhy	Msu	Mhf	Mhc	Ala	Pas
Genome (Mbp)	0.97	0.75–0.78	0.84–0.95	1.21	0.94	0.81	0.58	0.66	1.01	0.79	0.89	0.74	1.15	0.91	1.49	0.86
G+C content (%)	28.2	25	25–27	24	29.4	40	31.7	27.1	31	28	28	31.1	38.8	35.3	31	28
CDS	782	608	608	985	803	677	475	537	742	694	679	884	1545	1173	1380	754
Gene density (%)	82.5	95	93	83	89.5	88.7	90	89.8	91	91	88	89.9	94.2	92.8	90	73
Average CDS length (bp)	1013	1116	1010	982	1058	1011	1040	1107	1206	1058	1178	783	693	726	981	785
CDS with predicted function	470	407	378	581	526	333	323	345	469	464	412	293	299	286	1006	446
Hypothetical CDS	279	201	230	266	239	123	96	106	123	63	158	517	1246	887	NR	257
No. of tRNA genes	32	33	33	30	34	39	40	33	33	34	30	32	31	31	35	32
No. of rRNA genes																
16S	2	2	2	2	1	1	1	2	2	2	1	1	1	1	2	2
23S	2	2	2	2	1	1	1	2	2	2	1	1	1	1	2	2
5S	2	2	2	2	1	1	1	2	2	2	1	1	1	1	2	2

Udv = *U*. *diversum*, Upa = *U*. *parvum* (average between serotypes), Uua = *U*. *urealyticum* (average between serotypes), Mmy = *M*. *mycoides* subsp *mycoides*, Mbo = *M*. *bovis*, Mpn = *M*. *pneumoniae*, Mge = *M*. *genitalium*, Mho = *M*. *hominis*, Mgal = *M*. *galliseptcium*, Msy = *M*. *synoviae*, Mhy = *M*. *hyopneumoniae*, Msu = *M*. *suis*, Mhf = *M*. *haemofelis*, Mhc = *M*. *haemocanis*, Ala = *Acholeplasma laidlawii*, Pas = ‘Candidatus Phytoplasma asteris’. NR = not reported.

**Table 2:** Coding DNA sequences (CDSs) of *Ureaplasma diversum* ATCC 49782 genome classified by TIGR role category.

Name	Number	%
Unclassified	33	4.22%
Amino acid biosynthesis	0	0.00%
Purines, pyrimidines, nucleosides, and nucleotides	13	1.66%
Fatty acid and phospholipid metabolism	6	0.77%
Biosynthesis of cofactors, prosthetic groups, and carriers	9	1.15%
Central intermediary metabolism	8	1.02%
Energy metabolism	20	2.56%
Transport and binding proteins	73	9.34%
DNA metabolism	63	8.06%
Transcription	11	1.41%
Protein synthesis	85	10.87%
Protein fate	30	3.84%
Regulatory functions	4	0.51%
Signal transduction	3	0.38%
Cell envelope	55	7.03%
Cellular process	38	4.86%
Mobile and extrachromosomal element functions	8	1.02%
Unknown function	103	13.17%
Hypothetical proteins	279	35.68%

Comparisons among *U*. *diversum* ATCC 49782 and the human ureaplasma serovars (10 serovars of *U*. *urealyticum*, and 4 serovars of *U*. *parvum*) [21] indicate a larger genome size in *U*. *diversum* (*U*. *diversum*– 0.97 Mbp; *U*. *urealyticum*—0.84–0.95 Mbp; *U*. *parvum*—0.75–0.78 Mbp) and a slight higher G+C content when compared to other ureaplasmas (*U*. *diversum*—28.2%; *U*. *urealyticum*– 25–27%; *U*. *parvum*– 25%). *U*. *diversum* showed 782 CDSs; but *U*. *urealyticum* and *U*. *parvum* have an average of 608 CDSs. The hypothetical CDSs in *U*. *diversum* are 279, in *U*. *urealyticum* the average is 230 and in *U*. *parvum* the average is 201. These genetic differences may reflect the host specificity of *U*. *diversum*.

### Gene synteny and phylogeny

**Fig 2:**
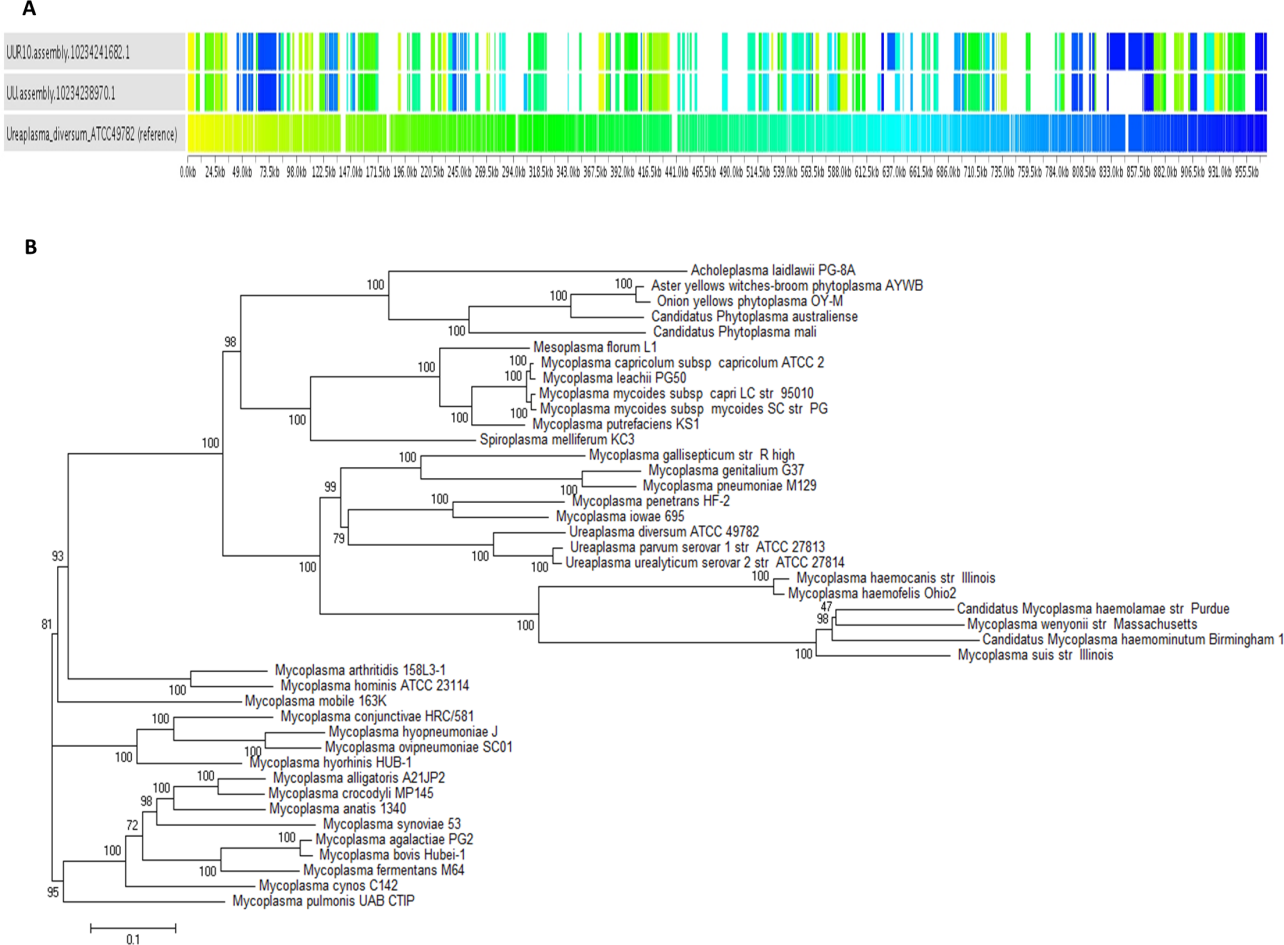
Gene synteny and phylogeny. (A) Syntenic maps of ureaplasma genomes. Sybil map used *U*. *diversum* ATCC 49782 as a reference genome. (B) Phylogenetic trees based on 32 concatenated proteins of *Mollicutes*.

In the present study, we used the gene synteny to compare the genomes of *U*. *diversum* and human ureaplasmas. CDSs that are conserved among the three species do not have the same organization, suggesting significant genomic reorganization (Fig 2A).

Moreover, phylogenetic trees based on concatenated protein sequences were constructed (Fig 2B). This protein concatenation approach has been frequently shown to increase resolution and robustness of phylogenetic analyses of mycoplasma species [22]. The phylogenetic relationship between *U*. *diversum* and human ureaplasmas remained the same compared with data from other studies with 16S rRNA and 16S-23S rRNA intergenic spacer [19, 20]. Ureaplasmas continue to branch within Mycoplasma clades [22]; the genus name Ureaplasma speaks to the fact that these organisms metabolize urea, and was coined before the era of DNA sequence based taxonomy. Interestingly, the phylogeny of *Ureaplasma* species corresponds to the evolutionary tree of the host animal species, suggesting coevolution between ureaplasmas and animals [20]. Although determinants for host ranges and susceptibilities to mycoplasma infections are currently unknown, *Ureaplasma* species have defined host ranges consisting of specific animal species [19].

### Metabolic pathways

**Fig 3:**
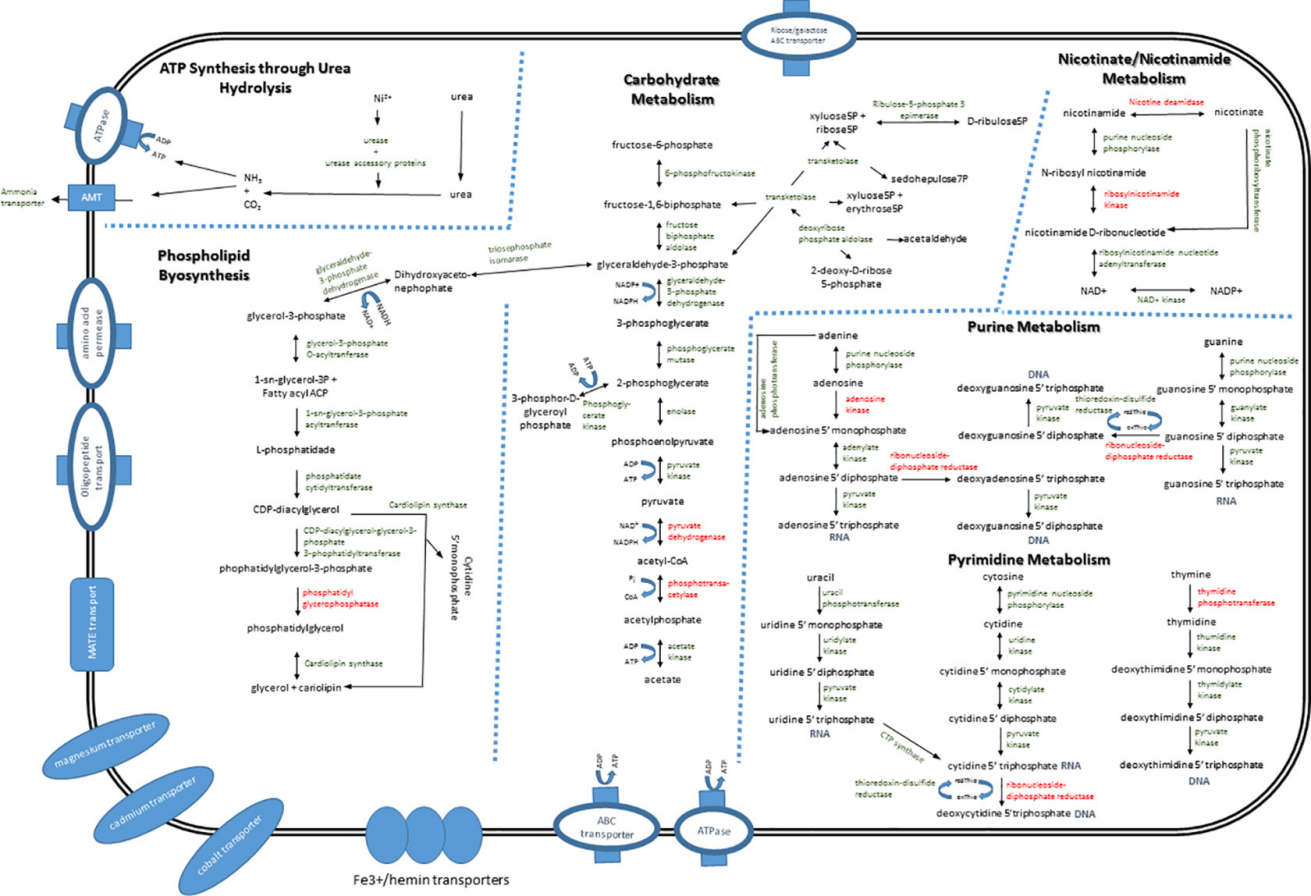
Metabolic map of *U*. *diversum* ATCC 49782. A view of the transporters and main metabolism pathways. Metabolic products are shown in black and ureaplasma proteins in green. Enzymes that were not found in *U*. *diversum* are shown in red.

Analyzing the predicted set of *U*. *diversum* CDSs, it was observed that the metabolic pathways are identical to that of other ureaplasmas (Fig 3). The main CDSs for the urease activity was annotated in the genome of *U*. *diversum* accordingly: urease (three-subunit urease + accessory proteins), an ammonia/ammonium transporter, and a F_O_F_1_-ATPase. Ureaplasma generates 95% of its ATP through the hydrolysis of urea by urease [23, 24]. Hydrolysis of urea generates an electrochemical gradient through accumulation of intracellular ammonia/ammonium. The gradient fosters a chemiosmotic potential that generates ATP. However, as in human ureaplasmas [25], nickel and urea transporters were not identified. Urea transporters are considered rare among bacteria and have been described in only few urease-positive organisms [26]. It is believed that urea can simply diffuse across membranes into the cytoplasm, but whether or not this is the mechanism used by *U*. *diversum* to acquire this metabolite from the environment is unknown.

The sources of the 5% of ureaplasma ATP production not from the urea hydrolysis are most probably obtained from substrate phosphorylation [25]. CDSs coding for enzymes of the Embden-Meyerhoff-Parnas (EMP, glycolysis) pathway were present, except for the glucose-6-phosphate isomerase, which catalyzes the step leading to fructose 6-phosphate production. The absence of the enzyme was also observed in human ureaplasmas [26, 27]. Thus, it is believed that an alternative enzyme may be acting for this pathway to be functional. The pyruvate metabolism in *U*. *diversum* is incomplete; the *U*. *diversum* genome does not contain orthologs for the pyruvate dehydrogenase complex. Therefore, the production of ATP from the oxidation of pyruvate to acetate is unlikely. Other mycoplasmas and ureaplasmas also do not contain this enzyme, but this *in vitro* activity has been described [27]. Moreover, the phosphotransferase system (PTS) in *U*. *diversum* is incomplete. Only two putative PTS components genes were annotated (a putative phosphotransferase enzyme IIB—gudiv_313; and a phosphotransferase enzyme family - gudiv_380). The PTS is found only in bacteria which catalyzes the transport and phosphorylation of numerous monosaccharides, disaccharides, amino sugars, polyols, and other sugar derivatives [28]. Thus, it is believed that the use of glucose may have been abolished in ureaplasmas. This can be an interesting evolutionary characteristic of genomic reduction. Glycolysis is likely used only to provide substrates for the PPP pathway and for the synthesis of glycerol.

As observed in other mycoplasma species as well as with *U*. *parvum* [25], the pentose phosphate pathway is incomplete, with the absence of CDSs encoding glucose-6-phosphate dehydrogenase and 6-phosphogluconate dehydrogenase. However, the pentose phosphate (PP) pathway in mycoplasmas is considered to be functional [29]. Therefore, this is most likely the case for ureaplasmas as well.

The Nicotinate/Nicotinamide metabolism appears to be incomplete but functional for NADPH generation. The presence of the enzyme NAD kinase was observed in *U*. *diversum*. This enzyme has been related to the interconversion of NAD+ and NADP+ in nicotinate/nicotinamide metabolism, which plays a critical role in maintaining the NADH/NADPH pool balance inside the bacterial cell [30].

As expected, CDSs for the *de novo* biosynthesis of purines or pyrimidines were not identified in *U*. *diversum* genome. We also did not identify the enzyme ribonucleoside diphosphate reductase responsible for the conversion of ribonucleosides to deoxyribonucleosides. Like *U*. *urealyticum*, *U*. *diversum* could import all its deoxyribonucleosides and/or deoxyribonucleoside precursors, or have a different mechanism for converting ribonucleosides to deoxyribonucleosides. Moreover, the absence of the enzyme thymidine phosphorylase suggests that thymidine is the precursor imported for dTTP production [30] or another enzyme, still unknown, could supply this function.

Mycoplasmas are thought to be completely incapable of fatty acid biosynthesis from acetyl-CoA, probably due to the loss of genetic material. Phospholipids, glycolipids and sterols are the three major lipid constituents of cell membranes. This pathway has been poorly described in mycoplasmas and not all enzymes are identified [29]. Ureaplasmas have been described in the past as capable of *de novo* synthesis of saturated and unsaturated fatty acids [31]; however, our metabolic network analyses identified a very limited number of known pathways related to fatty acid biosynthesis in *U*. *diversum*. Among the few identified pathways, metabolic reactions from glyceraldehyde-3-phosphate to cardiolipin are mostly preserved, but one enzyme is missing, the phosphatidyl glycerophosphatase. The role of this enzyme may be replaced by the enzyme cardiolipin synthase, which can convert cytidine 5' diphosphate diacylglycerol directly to cardiolipin in the presence of phosphatidylglycerol. Moreover, phospholipids are of exclusive bacterial origin, therefore excluding the possibility that it is provided by the serum lipids normally present in broth media, the only possibility is that it is synthesized [32].

### Transporters

Because of their genetic limitations, ureaplasmas must import more nutrients for cell growth than do most other bacteria. A total of 37 transporter-related CDSs were identified in *U*. *diversum* (Fig 3). ABC systems-related sequences represent 83.78% (31/37) of all transporter-related CDSs. Nonetheless, mycoplasmas have fewer transporters than other bacteria, and it is believe that these transporters have broader substrate specificities to compensate this limitation [32]. In *U*. *diversum*, a number of transport systems that are likely essential could not be found, such as bases/nucleotides, nickel and urea transporters. Urea transporters in bacteria are relatively rare. There are only three bacterial transporters classes, the ABC urea transporters, the Yut transporter, and the UreI transporter [33]. No homologous gene for these proteins was found in the genome of *U*. *diversum*. It is likely that CDSs of yet unknown function or other transporters are participating in the transport of such molecules.

### Virulence and pathogenicity mechanisms

**Fig 4:**
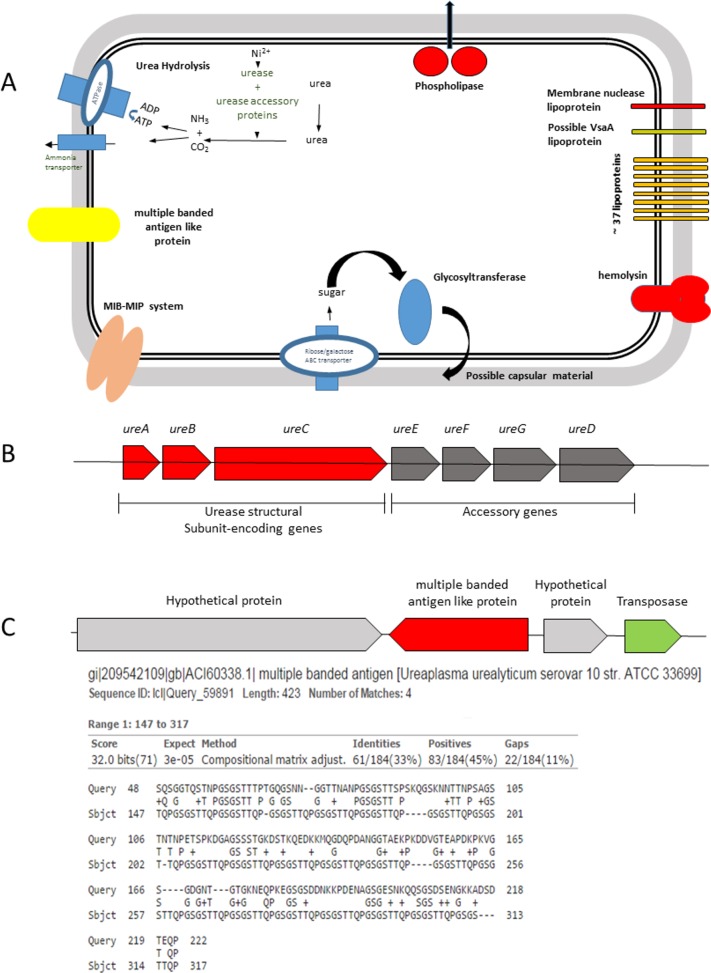
Virulence and pathogenicity mechanisms. (A) Virulence map of *U*. *diversum* ATCC 49782. (B) Schematic representation of the urease gene cluster from *U*. *diversum* ATCC 49782. Structural subunits: *ure*A (gudiv_255), *ure*B (gudiv_254), and *ure*C (gudiv_253). Accessory proteins *ure*E (gudiv_252), *ure*F (gudiv_251), *ure*G (gudiv_250), and *ure*D (gudiv_249) (C) Diagram of *Ureaplasma diversum* Multiple-Banded Antigen-like protein (MBA-like—gudiv_653) and locus and similarity of MBA-like with the human ureaplasmal Multiple-Banded Antigen (MBA) (Accession number: AF055358.2).

To date, only few features have been associated with virulence of *U*. *diversum*, such as the activity of urease and phospholipase, and its ability to invade some eukaryotic cells [8]. After analyzing the genome of *U*. *diversum*, it was possible to find CDSs that are likely related to bacterial pathogenicity and can expand our knowledge regarding the mechanisms by which *U*. *diversum* causes disease. CDSs of urease (gudiv_255, gudiv_254, gudiv_253, gudiv_252, gudiv_251, gudiv_250, and gudiv_249), phospholipase (gudiv_472), hemolysin (gudiv_91), and a Mycoplasma Ig binding protein (MIB)—Mycoplasma Ig protease (MIP) system (gudiv_161/gudiv_162 and gudiv_612/gudiv_613) were found and are described below in more detail. In addition, CDSs that are likely related to the production of a capsular structure (gudiv_216) and surface molecules were observed. A scheme of these virulence factors is found in Fig 4A.

#### Urease

In addition to the hydrolysis of urea, the enzyme urease can also be a virulence factor of ureaplasmas. The toxicity of urease is mediated by the amount of ammonium ions or free ammonia generated from urea [34]. The urease activity of human ureaplasmas produces ammonia, which can damage host tissues due to changes in the pH [35]. To date, there is no data in the literature on the pathogenic effects of the expression of the urease gene by *U*. *diversum*. Nevertheless, the analysis of the organization of the *U*. *diversum* urease gene cluster revealed the same organization of the human ureaplasma urease gene clusters [36]. There are three CDSs encoding structural subunits, *ureA* (gudiv_255), *ureB* (gudiv_254), and *ureC* (gudiv_253). Furthermore, downstream of *ureC*, four CDSs coding for accessory proteins (*ureE—*gudiv_252, *ureF*—gudiv_251, *ureG—*gudiv_250, and *ureD—*gudiv_249) were found (Fig 4B). A high identity of these CDSs when compared to CDSs of human ureaplasmas was also observed (above 90%).

#### Phospholipase

An array of potent hydrolytic enzymes has been identified in mycoplasmas, including phospholipases, proteases and nucleases [37]. Phospholipase C, A_1_, and A_2_ (PLC, PLA1, PLA2) activities have been reported in human ureaplasmas, however, no genes showed significant similarity to known sequences of PLC, PLA1, or PLA2 [38]. Orthologs to these CDS, however, were not found in the *U*. *diversum* genome. This is somewhat surprising, considering a recent report showing that *U*. *diversum* reference strains, including the ATCC 49782 sequenced herein, and clinical isolates have high phospholipase activity [8]. It has been speculated that this phospholipase activity could have helped the *in vitro* invasion of Hep-2 cells by *U*. *diversum* [8]. Furthermore, it has been shown that a similar phospholipase activity of *U*. *diversum* are able to interfere with prostaglandin E_2_ and prostaglandin F_2a_ production by bovine endometrial cells and induce premature labor [18]. In *U*. *diversum*, genes encoding phospholipase D family protein (PLD) (gudiv_472) and triacylglycerol lipase (Lipase family, such as phospholipases) (gudiv_748) were identified. Therefore, in contrast to human ureaplasmas, the PLD is likely the true virulence factor of *U*. *diversum* linked to cell invasion and premature labor in intrauterine infection, suggesting the phospholipase activity detected in our previous study [8] was PLD rather than phospholipase C activity. Experimental studies should be conducted with PLD to confirm this hypothesis.

#### Hemolysin

Hemolysins cause lysis of erythrocytes by forming pores of varying diameters in the host cell membrane [39]. There is one CDS (gudiv_91) coding a putative hemolysin protein in the genome of *U*. *diversum*. The predicted protein sequence has 63.1% identity with the hemolysin found in human ureaplasmas. In *U*. *parvum* serovar 3, this hemolysin has been characterized as an α-hemolysin (hlyA) with both hemolysin and cytotoxic activities [25]. Other mycoplasmas also have hemolysins [40, 41], but no orthologs to these proteins were found in the *U*. *diversum* genome. It seems that the mycoplasma or ureaplasma hemolysins belong to a unique class of bacterial toxins; more studies are needed to better understand the role of this enzyme in the pathogenesis of bovine ureaplasmas.

#### MIB-MIP system

Recently, a new virulence factor has been described in *Mollicutes*: the MIB-MIP system [42]. MIB acts as a high-affinity IgG binding protein, whereas MIP specifically cleaves the IgG heavy chain at an unconventional site, located between the VH and CH3 domains, and inactivate this molecule. This MIB–MIP system is encoded by a pair of genes often found in several copies in a wide range of mycoplasmas that infect various hosts [42]. Accordingly, two copies of the MIB-MIP system (gudiv_161/gudiv_162 and gudiv_612/gudiv_613) were found in the *U*. *diversum* genome. However, experimental studies should be conducted to better understand the role of this enzyme in the pathogenesis of bovine ureaplasmas.

#### Surface molecules

Although surface molecules of mycoplasmas are thought to play a crucial role in interactions with their hosts, very few had their biochemical function defined [43]. CDSs linked to lipoproteins (n = 37), multiple banded antigen like protein (MBA-like) (n = 1), membrane nuclease lipoprotein (n = 1) and variable surface antigen lipoproteins (possible VsA) (n = 1) were found in the genome of *U*. *diversum* (Fig 4A). Possibly due to the absence of a cell wall, mycoplasmas posses a larger number of membrane bound lipoproteins when compared to other eubacteria [44]. These molecules can act as virulence factors and/or be targets of humoral immunity [45]. They serve as potent cytokine inducers for monocytes/macrophages and have cytolytic activity [46]. This immunogenicity of lipoproteins is probably caused by their surface exposure and the presence of the amino-terminal lipoylated structure [47].

Among the detected lipoproteins, one CDS was identified as a membrane nuclease lipoprotein (gudiv_93). Membrane nuclease activity in mycoplasmas was first reported in *M*. *pulmonis* [48]. Nuclease activity in *Mollicutes* has been proposed as the mechanism by which these organisms acquire the precursors for nucleic acid production [49], but it can also function as a virulence factor. The characterization of nucleases in *Mollicutes* reveals that these enzymes differ from each other [50], and have been implicated in host cytotoxicity. The membrane nuclease MGA_0676, for example, is a remarkable pathogenic compound for the colonization and infection persistence of *M*. *gallisepticum* [51]. Additionally, *M*. *pneumoniae* nuclease (Mpn133) acts as a virulence determinant by binding to and internalizing within human airway cells [50].

Another important lipoprotein that has been identified is a putative variable surface antigen lipoprotein (gudiv_179) with 36% identity to the VsaA protein of *M*. *pulmonis*. Antigenic variation of surface proteins is thought to be a survival strategy for many mycoplasmas [52]. *M*. *bovis* possess a large family of variable surface lipoproteins designated as Vsps, which are encoded by 13 different genes. The Vsps undergo phase variation in the respiratory tract of infected calves, which limits the microorganism elimination [53]. The experiments with variable surface proteins support the hypothesis that the generation of antigenic variation in mycoplasmas is critical for the bacterial survival for extended periods within the host, but do not establish a link with disease severity [45].

Although with borderline similarity (33%) a multiple banded antigen like protein (MBA-like) was annotated in the genome of *U*. *diversum* ATCC 49782 (gudiv_653) (Fig 4C). This protein showed the conserved domain of the Multiple Banded Antigen-MBA of human ureaplasmas. The MBA is a surface exposed lipoprotein that can undergo size and phase variation *in vitro* and *in vivo* [54, 55]. The MBA is predicted to be a major human ureaplasma virulence factor and is the predominant antigen recognized by sera during infections in humans [56]. Analyzing the MBA locus composition among different human ureaplasmas [21], we can verify that the MBA-like locus in *U*. *diversum* features a similar organization with the MBA locus of *U*. *parvum* serovar 14 (UPA14) (Fig 4C). In particular, this area has two inverted hypothetical proteins and a transposase recognition site. Furthermore, unlike other ureaplasmas, in UPA14 and *U*. *diversum* loci, the DNA pol III alpha subunit is not located adjacent to the locus. More studies are needed to understand the biology and role of MBA-like in the pathogenicity of *U*. *diversum*. Interestingly, the MBA-like (gudiv_653) and the variable surface antigen lipoproteins (gudiv_179) are in the same paralogous gene family in *U*. *diversum*. These two proteins, added to fourteen other paralogous proteins (annotated as hypothetical proteins), may play a critical role in modulating the immune response against *U*. *diversum* (S1 Table).

#### Capsule

**Fig 5:**
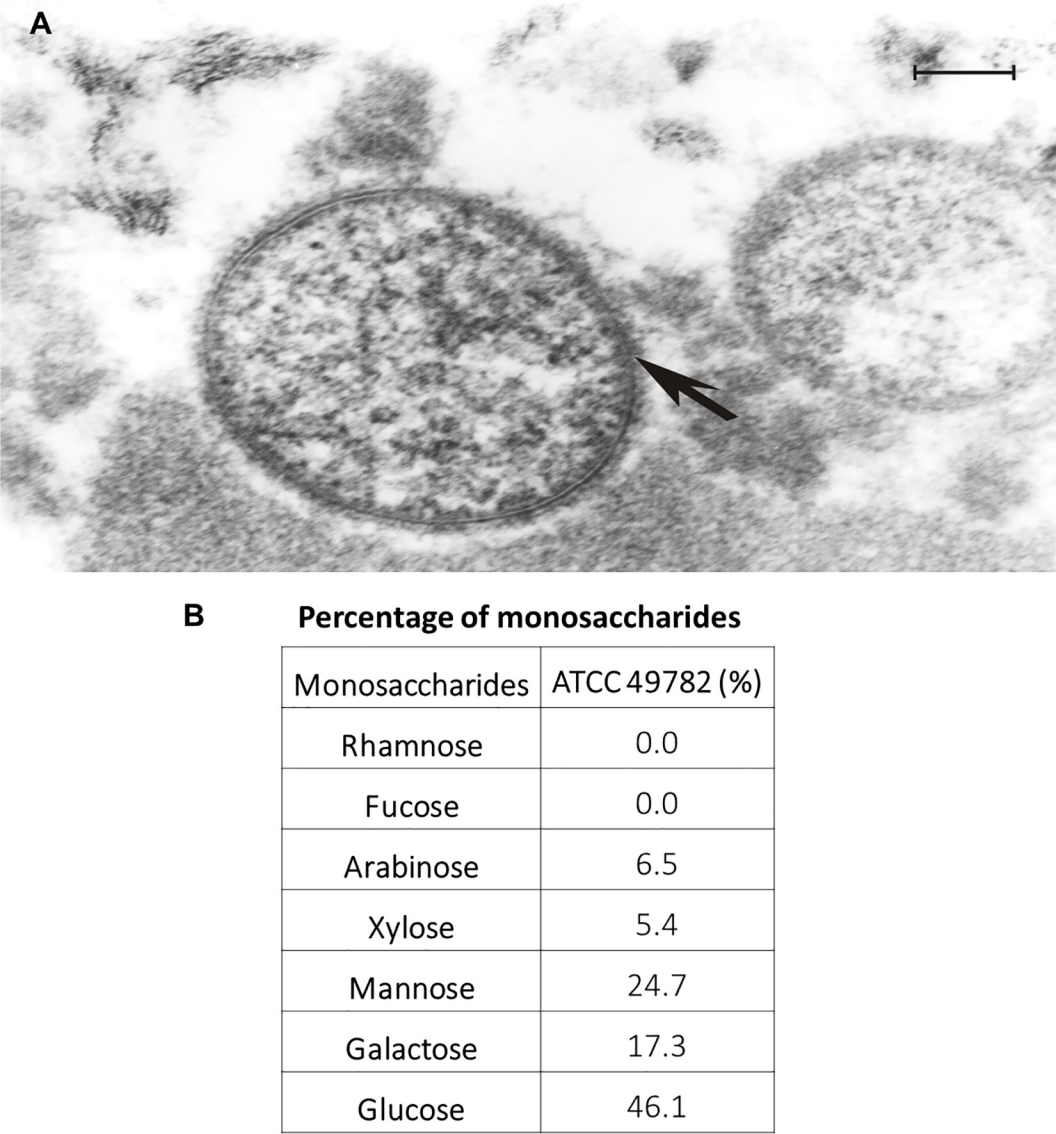
Capsule of *U*. *diversum*. (A) Electron microscopy of cells *U*. *diversum* ATCC 49782 obtained in the cultured isolates from mucovulvovaginal bovine semen and treated with red ruthenium dye, showing polysaccharide materials (electrodense external region indicated with arrowheads). Bar 100 nm. (B) Percentage of monosaccharides in capsular components of *U*. *diversum* ATCC 49782.

In the analysis of the genome of *U*. *diversum*, a glycosyltransferase enzyme (gudiv_216) was found [57]. Glycosyltransferase has also been described in the genome of human ureaplasmas. This enzyme is known to connect lipids and sugars that constitute the capsule of certain mycoplasmas [58]. In *M*. *mycoides* subsp. *mycoides*, the presence of glycosyltransferase is believed to be related to the attachment of galactan to the capsule [57]. Thus, we decided to evaluate if a capsular structure is also present in *U*. *diversum*. By using electron microscopy and the red ruthenium dye, a polysaccharide capsule extending from 11 to 17 nm outside of *U*. *diversum* cells was observed for the first time (Fig 5A). In a previous study with *U*. *urealyticum*, using the same dye utilized herein, a capsular structure was also observed, indicating that this may be a common feature of *Ureaplasma* species [13]. Increased virulence and adhesion to host cells are associated with the presence of a capsule in certain bacterial species [59]. For instance, the *M*. *gallisepticum* and *M*. *hyopneumoniae* capsules have been suggested as additional structures that assist the cytoadherence to host cells [60] in addition to the “bleb” (a mycoplasma structure containing adhesins and accessory proteins) [61]. In *M*. *mycoides* subsp. *mycoides* strains, the presence of a polysaccharide capsule causes host injury [62]. Other studies have correlated the presence of a capsule and the resistance of mycoplasma to phagocytic cells. In *M*. *dispar*, the capsule has been considered the most important structure to interfere the phagocytosis [63]. Therefore, the detection of a capsular structure in *U*. *diversum* opens venues for new studies related to the virulence and pathogenicity of this bacterium.

The chemical composition of the capsular material of *U*. *diversum* ATCC 49782 was subsequently evaluated. Arabinose, xylose, mannose, galactose and glucose were the main components detected in the capsular material of this bacterium (Fig 5B). The biochemical composition of capsular polymers of *Mollicutes* has been little studied. The capsules of *M*. *mycoides* subsp. *mycoides* SC, *M*. *mycoides* subsp. *capri* and *M*. *dispar* are the only ones described in the literature and are mainly composed of 1,6-galactose, polyglucan, and galacturonic acid polymers, respectively [64]. Probably, the capsular biosynthesis of *M*. *mycoides* subsp. *mycoides* is related to a glucose-dependent synthetic pathway due to the presence of a UDP-glucose 4-epimerase (GalE) and a glucose-1-phosphate uridylyltransferase (GalU) that uses glucose-1-phosphate to generate UDP-glucose [57]. In our *in silico* analysis, we did not observe genes related to the biosynthesis of polysaccharides. However, the presence of two genes of a sugar transporter was observed (Ribose/galactose ABC transporter—gudiv_307 and gudiv_308). We believe it is likely that these sugars are captured by *U*. *diversum* and are incorporated using glycosyltransferase to form the capsule.

#### Host immune response against surface molecules

**Fig 6:**
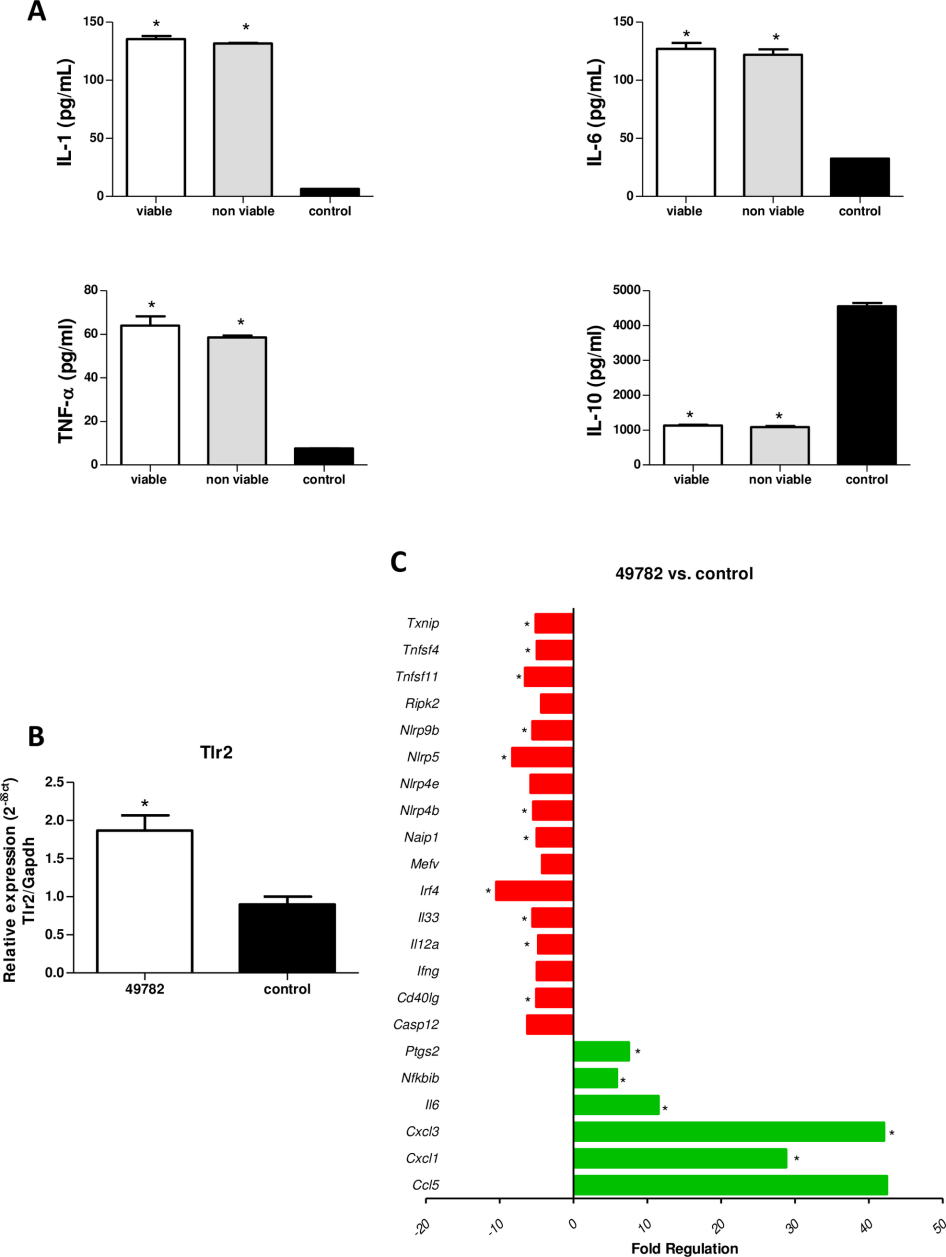
Host immune response against surface molecules. (A) IL-1β, IL-6, TNF-α and IL-10 levels in supernatant of macrophage J774 culture after infection with viable and nonviable *U*. *diversum* ATCC 49782 strain compared to uninfected cells. Statistical significance (p<0.05) is represented by the asterisk (*) (non-parametric Mann-Whitney analysis—One-tailed test, GraphPad Prism® version 6.01). (B) Gene expression of Toll-like receptors 2 in macrophage J774 culture after infection with *U*. *diversum* ATCC 49782 strain compared to the uninfected cells. Statistical significance (p<0.05) is represented by the asterisk (*) (non-parametric Mann-Whitney analysis—One-tailed test, GraphPad Prism® version 6.01). (C) up-regulated (green) and down-regulated genes (red) of the inflammasome pathways in macrophage J774 culture after infection with *U*. *diversum* ATCC 49782 strain compared to uninfected cells. Statistical significance (p<0.05) is represented by the asterisk (*) (non-parametric Mann-Whitney analysis—One-tailed test, GraphPad Prism® version 6.01).

In order to better understand the immune response against *U*. *diversum* surface molecules, we infect murine macrophages J774 with viable and nonviable microorganisms. In the analysis of cytokine production, there was an increased production of pro-inflammatory cytokines (IL-1β, IL-6, TNF-α) in both conditions compared to the negative control (p<0.05), but there was no statistical difference between the profile triggered by the viable and nonviable ureaplasma (Fig 6A). Therefore, it is suggested that surface molecules are the main determinants of immune system activation in *U*. *diversum*. Surface molecules are composed of bacterial pathogen-associated molecular patterns (PAMPs) that, when recognized by the innate immune system, trigger the production of pro-inflammatory cytokines from manifold cells, causing inflammation [65–67]. The ability of surface molecules of *Mollicutes* to induce the secretion of inflammatory cytokines such as IL-1β, TNF-α and IL-6 have been reported in studies with different species, e.g. *U*. *diversum* [5], *U*. *urealyticum* [68, 69], and *M*. *fermentans* [65].

The expression of Toll-like receptors (TLRs) and inflammasome pathway genes were also evaluated using murine macrophages J774 infected with viable *U*. *diversum* cells. It can be observed that *U*. *diversum* induced a higher gene expression of TLR2 when compared to the uninfected cells (Fig 6B) (p<0.05). The gene expression of other TLRs are presented in S1 Fig. The downregulation of TLR5, TLR8, TLR9 and TLR11 was als observed. These findings are supported by the literature, which indicates that TLR2 is the main receptor for the immune response of *Mollicutes* [45]. However, a study of *U*. *urealyticum* infecting macrophages demonstrated the activation of TLR2 and TLR4 [70]. Moreover, the absence of TLR 2 was related to the not recognition of bacterial lipoproteins of *M*. *fermentans* [71, 72].

The results of the inflammasome expression assay demonstrate the up-regulation of genes related to chemokine binding domains, interleukin-6, inhibitors of kinases and B cells, NF-kB, and interferon regulatory prostaglandin endoperoxide (Fig 6C). In contrast, caspase 12, CD40 ligand, interferon gamma, interleukin 12A, and 33 families of ligand to NLR and tumor necrosis factor genes were downregulated. Several studies have demonstrated the importance of the inflammasomes in response to mycoplasma infection [73]; however, the literature does not define mechanisms involved in the inflammasome response against *U*. *diversum*. A study of the development of gastric tumors using monocytes exposed to *M*. *hyorhinis* confirmed a high production of IL-1β and IL-18-induced NLRP3-dependent mechanisms [74]. It has been shown that monocytes and macrophages infected with live or inactivated by heat *Acholeplasma laidlawii* have the capacity to promote the release of IL-1β by the presence of cytosolic ASC (apoptosis-associated speck-like protein containing a carboxy-terminal CARD), promoting inflammasome activation [75]. In the present study, the up-regulation of NLRPs was not observed. Therefore, more studies are necessary to elucidate the involvement of inflammasome activation in the immune response induced by *U*. *diversum*. This study is the first report of the activation mechanisms involved in the immune response against *Ureaplasma diversum*. These findings can contribute to the studies on macrophage activation for the regulated secretion of cytokines to prevent tissue damage caused by an exacerbated immune response. The results described herein will also support further research to understand the possible effector mechanisms that lead to effective protection against *U*. *diversum* infection.

## Conclusion

The whole genome sequence described herein represents a valuable new resource for the study of *U*. *diversum* on a genetic basis. Furthermore, extensive analyses of these data helped us to detect important biological features of this mollicute and its mechanisms of pathogenicity. The results of this study will help to better understand this microbe, and can direct future research on the pathogenicity of this bacterium in cattle. Moreover, it will also aid the development of new strategies for treatment, prevention and control of ureaplasma infections.

## Material and Methods

### Bacterial strain culture conditions and DNA extraction

*Ureaplasma diversum* ATCC 49782 was originally isolated from a cow with clinically acute granular vulvitis in 1978 [10]. The virulence of this strain has been confirmed by many studies [1–5, 7, 8, 76]. *U*. *diversum* ATCC 49782 was first cultured in 2 mL of ureaplasma medium (UB) at 37°C, followed by propagation in 3,000 mL of the same broth. At the logarithmic growth phase (based on colorimetric changes), the culture was centrifuged at 20,600 x g for 30 minutes at 25°C. The DNA was extracted using a PureLink™ Genomic DNA Mini Kit (Life Technologies, Brazil) following the manufacture’s instructions.

### DNA sequencing, sequence assembly, gap closure and validation

The whole genome was sequenced from a paired-end library using Illumina HiSeq 2000 (Illumina, Inc., San Diego, CA) at the Purdue University Genomics Core Facility. Average reads of about 100 bases were assembled using ABySS 1.2.7. After assembly resulting from a 4,552X genome coverage, eight remaining gaps were closed using conventional PCR, followed by sequencing using Sanger method in both directions. The genome sequence of *U*. *diversum* strain ATCC 49782 has been deposited in the GenBank^®^ database under the accession no. CP009770.

### Identification of CDS and annotation

First-pass annotation was achieved using the NCBI (National Center for Biotechnology Information) prokaryotic genome annotation pipeline (PGAP). An initial set of CDSs was identified using PRODIGAL [77] and analyzed using the annotation engine MANATEE from the Institute for Genomic Sciences (University of Maryland, School of Medicine, Baltimore, MD) [78]. Individual CDS were manually curated; evidence generated by the pipeline (including BER, HMMs, PROSITE matches, TMHMM, and SignalP) was used to infer annotations. CDSs with an HMM score below the trusted value, less than 40% identity, or only local similarities to known protein sequences were called hypothetical proteins. tRNAs were located using tRNA-scan-SE [79]. Lipoproteins and signal peptides were identified using LipoP and SignalP algorithms, respectively [80, 81], and paralogous gene families were recognized using BLASTCLUST analysis (National Center for Biotechnology Information, NCBI, Bethesda, MD) using 30% sequence identity and 70% covered length thresholds. Comparative analyses with other bacterial genomes were performed based on genome annotations deposited in the NCBI Genome database. Considering that the genome annotation of human ureaplasmas deposited in GenBank, in particular of *U*. *parvum* serovars 3, may be outdated (*U*. *parvum* serovars 3 genome was one of the first bacterial genomes to be sequenced and annotated), we used tblastn to search for possible misannotated sequences when an ortholog was not found. A diagram of the overall structure of genome was generated using a DNAPlotter version 1.4 from Artemis 12.0, Sanger Institute [82].

### Comparative and phylogenetic analyses

Whole genome synteny comparisons among *U*. *diversum*, *U*. *urealyticum* and *U*. *parvum* were performed using Sybil [83]. In Sybil, orthologs (homologous protein sequences from different bacterial species) were used to define syntenic relationships between species/strains.

Phylogenetic analyses of the 41 mollicutes were also performed using a multiple sequence alignment of 32 concatenated protein sequences from each organism (S2 Table). These proteins were chosen based on previous reports of phylogenomic analyses in prokaryotes [84, 85] according to their presence in all selected species, absence of additional fused domains, no subjection to HGT, and completeness [84]. Following protein concatenation using the UNION tool from EMBOSS [86], the sequences were aligned using MAFFT version 7 [87]. The resulting alignment was employed to build a phylogenetic tree using the neighbor-joining method [88] and maximum likelihood, with 1,000 bootstrap replicates from MEGA 6 [89].

### Capsule detection

The ureaplasma was cultured in 50 mL of UB media. Cells in the logarithmic growth phase were collected by centrifugation at 20,600 x g for 30 minutes, and the pellets were suspended in 3% of glutaraldehyde and 0.2% of ruthenium red in 0.1M-cacodylate buffer (pH 7.4). Ruthenium red is a polycationic dye that binds polysaccharides and is frequently used to detect bacterial capsules [59]. After five washes with 0.05 M cacodylate buffer, the ureaplasmas were post-fixed in 1% (w/v) osmium tetroxide, then dehydrated in a graded series of ethanol and embedded in Spurr resin (Electron Microscopy Sciences, Fort Washington, PA, USA). Ultrathin sections collected on 200-mesh copper grids were stained with uranyl acetate and lead citrate before examination in a JEOL 1010 Transmission Electron Microscope.

To extract and purify the capsular component, the bacterial strain was cultured in 2 liters of UB media and then centrifuged (20,600 xg for 50 minutes) to retain the pellet. The pellet was washed twice in PBS (137 mM NaCl; 2.7 mM KCl; 4.3 mM Na_2_HPO_4_; 1.47 mM KH2PO_4_) (to avoid contamination of the polysaccharides derived from the culture medium), and resuspended in 10 mL of the same buffer. The extraction of capsular polysaccharide continued by the phenol-chloroform method [64]. To assess the monosaccharide composition, each polysaccharide was hydrolyzed with 300 μL of 2 mol L^-1^ of trifluoroacetic acid, at 100°C for 8 h. The samples were dried under nitrogen stream and then dissolved in water 300 μL and the pH was elevated to 8 by addition of NH_4_OH. The samples were reduced with NaBH_4_ at room temperature for 4 hours, then the cationic resin was added to remove salts, the samples were dried under nitrogen stream and followed by dissolution/dryness process in methanol (500 μL, x 3) to remove the residual borate. The alditol products were acetylated by addition of 200 μL of acetic anhydride-pyridine (1:1, v/v), held overnight at room temperature. Methanol (200 μL) was added to stop reaction and the samples were dried under nitrogen stream. The alditol acetates were analyzed by gas chromatography coupled to mass spectrometry (GC-MS—Varian, model Saturn 2000) with Ion Trap analyzer. The chromatography was developed in a DB-225-MS fused silica capillary column (30 m, 0.25 mm, i.d. and 0.25 m of film thickness). The temperatures were: injector 250°C and the oven programmed from 50°C to 220°C at 40°C min^-1^. The monosaccharides (as alditol acetates) were identified on the basis of their mass spectra at electron ionization (EI, 70eV) and authentic standards (Sigma-Aldrich).

### Macrophage interaction

Inoculations in murine macrophages J774 (ATCC® TIB-67™) were performed with viable bacterial strain and bacterial strain inactivated by heat (100°C for 30 minutes) for 24 hours (MOI 1:100). The inactivation was confirmed by absence of a positive culture on ureaplasma medium (UB). Negative controls without bacteria inoculation were also used. After 24 hours, the supernatant of the bottles was collected and frozen at -80°C to perform the ELISA. The cells were washed with trypsin and the cell suspension was placed in microtubes with RNA later for RNA extraction procedures.

The dosage of the cytokines TNF-α, IL-1β, IL-6, IL-10 was set using Ready-SET-GO enzyme-linked immunosorbent assay kit (eBioscience, San Diego, USA). The mRNA from cells was extracted using TRIzol Plus RNA purification kit (Invitrogen, USA), following the protocol supplied by the manufacturer. The cDNA was obtained using a retro-transcription (RT) from the mRNA using the SuperScript ® III Reverse Transcriptase kit. cDNA was used in a qPCR reaction to determine gene expression of TLR's 1–9 and 11 according to a previously described protocol [90]. The RT-qPCR was run three independent times with at least five samples per group. Analysis of relative gene expression data was performed using the 2(-Delta Delta C(T)) Method [91]. GAPDH served as housekeeping gene to assess the overall cDNA content.

Gene expression of the inflammasome pathway was verified by quantitative PCR (qPCR) methodology. The cDNA obtained was subjected to analysis using Mouse Inflammasomes RT^2^ Profiler™ qPCR Array kit (Qiagen-SABioscience, Brazil) for the expression of 84 key genes involved in the function of inflammasomes, protein complexes involved in innate immunity, as well as general NOD-like receptor (NLR) signaling (S3 Table). This array includes genes encoding inflammasome components as well as genes involved in downstream signaling and inhibition of the inflammasome function. In addition, this array includes other NLR family members, which may potentially form additional inflammasomes and their downstream signaling genes. All procedures were performed according to the manufacturer’s instructions.

## Supporting Information

S1 Fig Gene expression of Toll-like receptors in macrophage J774 culture after infection with *U*. *diversum* ATCC 49782 strain compared to uninfected cells. Statistical significance (p<0.05) is represented by the asterisk (*) (non-parametric Mann-Whitney analysis—One-tailed test, GraphPad Prism® version 6.01).
(TIF)

S1 Table Analyses of paralogous gene families in *Ureaplasma diversum* ATCC49782. (DOCX)

S2 Table Cluster of orthologous groups (COG)/proteins used for phylogenetic analysis. (DOCX)

S3 Table Genes evaluated using Mouse Inflammasomes RT^2^ Profiler™ qPCR Array kit. (DOCX)

## References

[1] Amorim A, Marques L, Santos AM, Martins H, Barbosa M, Rezende I, et al Apoptosis in HEp-2 cells infected with Ureaplasma diversum. Biological research. 2014;47(1):38 10.1186/0717-6287-47-38 25299837 PMC4167145

[2] Britton AP, Miller RB, Ruhnke HL, Johnson WM. Protein A gold identification of ureaplasmas on the bovine zona pellucida. Can J Vet Res. 1989;53(2):172–5. 2469532 PMC1255543

[3] Britton AP, Ruhnke HL, Miller RB, Johnson WH, Leslie KE, Rosendal S. In vitro exposure of bovine morulae to Ureaplasma diversum. Can J Vet Res. 1987;51(2):198–203. PubMed Central PMCID: PMC1255303. 3607652 PMC1255303

[4] Buzinhani M, Yamaguti M, Oliveira RC, Cortez BA, Marques LM, Machado-Santelli GM, et al Invasion of Ureaplasma diversum in bovine spermatozoids. BMC research notes. 2011;4:455 10.1186/1756-0500-4-455 PubMed Central PMCID: PMC3219583. 22032232 PMC3219583

[5] Chelmonska-Soyta A, Miller RB, Ruhnke L, Rosendal S. Activation of murine macrophages and lymphocytes by Ureaplasma diversum. Can J Vet Res. 1994;58(4):275–80. PubMed Central PMCID: PMC1263712. 7889459 PMC1263712

[6] Hobson N, Chousalkar KK, Chenoweth PJ. Ureaplasma diversum in bull semen in Australia: its detection and potential effects. Australian veterinary journal. 2013;91(11):469–73. 10.1111/avj.12113 . 24571302

[7] Kreplin CM, Ruhnke HL, Miller RB, Doig PA. The effect of intrauterine inoculation with Ureaplasma diversum on bovine fertility. Can J Vet Res. 1987;51(4):440–3. 3453263 PMC1255361

[8] Marques LM, Ueno PM, Buzinhani M, Cortez BA, Neto RL, Yamaguti M, et al Invasion of Ureaplasma diversum in Hep-2 cells. BMC microbiology. 2010;10:83 10.1186/1471-2180-10-83 20236540 PMC2907839

[9] Miller RB, Ruhnke HL, Doig PA, Poitras BJ, Palmer NC. The effects of Ureplasma diversum inoculated into the amniotic cavity in cows. Theriogenology. 1983;20(3):367–74. 0093-691X(83)90071-7 [pii]. . 16725853 10.1016/0093-691x(83)90071-7

[10] Ruhnke HL, Doig PA, MacKay AL, Gagnon A, Kierstead M. Isolation of Ureaplasma from bovine granular vulvitis. Can J Comp Med. 1978;42(2):151–5. 352491 PMC1277608

[11] Ruhnke HL, Palmer NC, Doig PA, Miller RB. Bovine abortion and neonatal death associated with Ureaplasma diversum. Theriogenology. 1984;21(2):295–301. 0093-691X(84)90415-1 [pii]. . 16725880 10.1016/0093-691x(84)90415-1

[12] Buzinhani M, Yamaguti M, Oliveira RC, Cortez BA, Miranda Marques L, Machado-Santelli GM, et al Invasion of Ureaplasma diversum in bovine spermatozoids. BMC research notes. 2011;4(1):455 1756-0500-4-455 [pii] 10.1186/1756-0500-4-455 . 22032232 PMC3219583

[13] Howard CJ, Gourlay RN, Brownlie J. The virulence of T-mycoplasmas, isolated from various animal species, assayed by intramammary inoculation in cattle. The Journal of hygiene. 1973;71(1):163–70. 4632825 10.1017/s0022172400046337 PMC2130429

[14] Marques LM, Buzinhani M, Guimaraes AM, Marques RC, Farias ST, Neto RL, et al Intraspecific sequence variation in 16S rRNA gene of Ureaplasma diversum isolates. Veterinary microbiology. 2011;152(1–2):205–11. 10.1016/j.vetmic.2011.04.007 . 21601382

[15] Marques LM, Ueno PM, Buzinhani M, Cortez BA, Neto RL, Yamaguti M, et al Invasion of Ureaplasma diversum in Hep-2 cells. BMC microbiology. 2010;10:83 1471-2180-10-83 [pii] 10.1186/1471-2180-10-83 20236540 PMC2907839

[16] Silva JR, Ferreira LF, Oliveira PV, Nunes IV, Pereira IS, Timenetsky J, et al Intra-uterine experimental infection by Ureaplasma diversum induces TNF-alpha mediated womb inflammation in mice. Anais da Academia Brasileira de Ciencias. 2016 10.1590/0001-3765201620150244 . 26871498

[17] Buzinhani M, Buim MR, Yamaguti M, Oliveira RC, Mettifogo E, Timenetsky J. Genotyping of Ureaplasma diversum isolates using pulsed-field electrophoresis. Veterinary journal. 2007;173(3):688–90. S1090-0233(06)00039-6 [pii] 10.1016/j.tvjl.2006.02.001 . 16616531

[18] Kim JJ, Quinn PA, Fortier MA. Ureaplasma diversum infection in vitro alters prostaglandin E2 and prostaglandin F2a production by bovine endometrial cells without affecting cell viability. Infection and immunity. 1994;62(5):1528–33. 8168914 10.1128/iai.62.5.1528-1533.1994 PMC186347

[19] Harasawa R, Cassell GH. Phylogenetic analysis of genes coding for 16S rRNA in mammalian ureaplasmas. Int J Syst Bacteriol. 1996;46(3):827–9. . 8782697 10.1099/00207713-46-3-827

[20] Harasawa R, Lefkowitz EJ, Glass JI, Cassell GH. Phylogenetic analysis of the 16S-23S rRNA intergenic spacer regions of the genus Ureaplasma. The Journal of veterinary medical science / the Japanese Society of Veterinary Science. 1996;58(3):191–5. . 8777224 10.1292/jvms.58.191

[21] Paralanov V, Lu J, Duffy LB, Crabb DM, Shrivastava S, Methe BA, et al Comparative genome analysis of 19 Ureaplasma urealyticum and Ureaplasma parvum strains. BMC microbiology. 2012;12:88 10.1186/1471-2180-12-88 22646228 PMC3511179

[22] Guimaraes AM, Santos AP, do Nascimento NC, Timenetsky J, Messick JB. Comparative genomics and phylogenomics of hemotrophic mycoplasmas. PloS one. 2014;9(3):e91445 10.1371/journal.pone.0091445 24642917 PMC3958358

[23] Blanchard A, Razin S, Kenny GE, Barile MF. Characteristics of Ureaplasma urealyticum urease. Journal of bacteriology. 1988;170(6):2692–7. 3131306 10.1128/jb.170.6.2692-2697.1988 PMC211190

[24] Smith DG, Russell WC, Ingledew WJ, Thirkell D. Hydrolysis of urea by Ureaplasma urealyticum generates a transmembrane potential with resultant ATP synthesis. Journal of bacteriology. 1993;175(11):3253–8. 8501029 10.1128/jb.175.11.3253-3258.1993 PMC204721

[25] Glass JI, Lefkowitz EJ, Glass JS, Heiner CR, Chen EY, Cassell GH. The complete sequence of the mucosal pathogen Ureaplasma urealyticum. Nature. 2000;407(6805):757–62. 10.1038/35037619 . 11048724

[26] Glass JI. Ureaplasma urealyticum: an opportunity for combinatorial genomics. Trends in microbiology. 2001;9(4):163 . 11357828 10.1016/s0966-842x(01)01951-5

[27] Pollack JD. Ureaplasma urealyticum: an opportunity for combinatorial genomics. Trends in microbiology. 2001;9(4):169–75. . 11286881 10.1016/s0966-842x(01)01950-3

[28] Deutscher J, Francke C, Postma PW. How phosphotransferase system-related protein phosphorylation regulates carbohydrate metabolism in bacteria. Microbiol Mol Biol Rev. 2006;70(4):939–1031. 10.1128/MMBR.00024-06 17158705 PMC1698508

[29] Guimaraes AM, Santos AP, SanMiguel P, Walter T, Timenetsky J, Messick JB. Complete genome sequence of Mycoplasma suis and insights into its biology and adaption to an erythrocyte niche. PloS one. 2011;6(5):e19574 10.1371/journal.pone.0019574 21573007 PMC3091866

[30] Santos AP, Guimaraes AM, do Nascimento NC, Sanmiguel PJ, Martin SW, Messick JB. Genome of Mycoplasma haemofelis, unraveling its strategies for survival and persistence. Veterinary research. 2011;42:102 10.1186/1297-9716-42-102 21936946 PMC3196708

[31] Romano N, Rottem S, Razin S. Biosynthesis of saturated and unsaturated fatty acids by a T-strain mycoplasma (Ureaplasma). Journal of bacteriology. 1976;128(1):170–3. 977538 10.1128/jb.128.1.170-173.1976 PMC232840

[32] Yus E, Maier T, Michalodimitrakis K, van Noort V, Yamada T, Chen WH, et al Impact of genome reduction on bacterial metabolism and its regulation. Science. 2009;326(5957):1263–8. 10.1126/science.1177263 . 19965476

[33] Sachs G, Kraut JA, Wen Y, Feng J, Scott DR. Urea transport in bacteria: acid acclimation by gastric Helicobacter spp. The Journal of membrane biology. 2006;212(2):71–82. 10.1007/s00232-006-0867-7 . 17264989

[34] Ligon JV, Kenny GE. Virulence of ureaplasmal urease for mice. Infection and immunity. 1991;59(3):1170–1. 1997418 10.1128/iai.59.3.1170-1171.1991 PMC258383

[35] Kokkayil P, Dhawan B. Ureaplasma: current perspectives. Indian journal of medical microbiology. 2015;33(2):205–14. 10.4103/0255-0857.154850 . 25865969

[36] Neyrolles O, Ferris S, Behbahani N, Montagnier L, Blanchard A. Organization of Ureaplasma urealyticum urease gene cluster and expression in a suppressor strain of Escherichia coli. Journal of bacteriology. 1996;178(9):2725 8626347 10.1128/jb.178.9.2725-2725.1996 PMC178004

[37] Rottem S. Interaction of mycoplasmas with host cells. Physiological reviews. 2003;83(2):417–32. 10.1152/physrev.00030.2002 . 12663864

[38] DeSilva NS, Quinn PA. Characterization of phospholipase A1, A2, C activity in Ureaplasma urealyticum membranes. Molecular and cellular biochemistry. 1999;201(1–2):159–67. . 10630635 10.1023/a:1007082507407

[39] Goebel W, Chakraborty T, Kreft J. Bacterial Hemolysins as Virulence Factors. A Van Leeuw J Microb. 1988;54(5):453–63. 10.1007/Bf00461864 . 3144241

[40] Chambaud I, Heilig R, Ferris S, Barbe V, Samson D, Galisson F, et al The complete genome sequence of the murine respiratory pathogen Mycoplasma pulmonis. Nucleic acids research. 2001;29(10):2145–53. 11353084 10.1093/nar/29.10.2145 PMC55444

[41] Li Y, Zheng H, Liu Y, Jiang Y, Xin J, Chen W, et al The complete genome sequence of Mycoplasma bovis strain Hubei-1. PloS one. 2011;6(6):e20999 10.1371/journal.pone.0020999 21731639 PMC3120828

[42] Arfi Y, Minder L, Di Primo C, Le Roy A, Ebel C, Coquet L, et al MIB-MIP is a mycoplasma system that captures and cleaves immunoglobulin G. Proceedings of the National Academy of Sciences of the United States of America. 2016;113(19):5406–11. 10.1073/pnas.1600546113 27114507 PMC4868467

[43] Masukagami Y, Tivendale KA, Mardani K, Ben-Barak I, Markham PF, Browning GF. The Mycoplasma gallisepticum virulence factor lipoprotein MslA is a novel polynucleotide binding protein. Infection and immunity. 2013;81(9):3220–6. 10.1128/IAI.00365-13 23798535 PMC3754236

[44] You XX, Zeng YH, Wu YM. Interactions between mycoplasma lipid-associated membrane proteins and the host cells. Journal of Zhejiang University Science B. 2006;7(5):342–50. 10.1631/jzus.2006.B0342 16615163 PMC1462930

[45] Browning GF, Marenda MS, Noormohammadi AH, Markham PF. The central role of lipoproteins in the pathogenesis of mycoplasmoses. Veterinary microbiology. 2011;153(1–2):44–50. 10.1016/j.vetmic.2011.05.031 . 21684094

[46] Zuo LL, Wu YM, You XX. Mycoplasma lipoproteins and Toll-like receptors. Journal of Zhejiang University Science B. 2009;10(1):67–76. 10.1631/jzus.B0820256 19198025 PMC2613965

[47] Chambaud I, Wroblewski H, Blanchard A. Interactions between mycoplasma lipoproteins and the host immune system. Trends in microbiology. 1999;7(12):493–9. 10.1016/S0966-842x(99)01641-8 . 10603485

[48] Minion FC, Goguen JD. Identification and preliminary characterization of external membrane-bound nuclease activities in Mycoplasma pulmonis. Infection and immunity. 1986;51(1):352–4. 3941002 10.1128/iai.51.1.352-354.1986 PMC261110

[49] Minion FC, Jarvill-Taylor KJ, Billings DE, Tigges E. Membrane-associated nuclease activities in mycoplasmas. Journal of bacteriology. 1993;175(24):7842–7. 8253673 10.1128/jb.175.24.7842-7847.1993 PMC206960

[50] Somarajan SR, Kannan TR, Baseman JB. Mycoplasma pneumoniae Mpn133 is a cytotoxic nuclease with a glutamic acid-, lysine- and serine-rich region essential for binding and internalization but not enzymatic activity. Cellular microbiology. 2010;12(12):1821–31. 10.1111/j.1462-5822.2010.01513.x 20690923 PMC3013625

[51] Xu J, Teng D, Jiang F, Zhang Y, El-Ashram SA, Wang H, et al Mycoplasma gallisepticum MGA_0676 is a membrane-associated cytotoxic nuclease with a staphylococcal nuclease region essential for nuclear translocation and apoptosis induction in chicken cells. Applied microbiology and biotechnology. 2015;99(4):1859–71. 10.1007/s00253-014-6185-6 . 25363559

[52] Noormohammadi AH, Markham PF, Kanci A, Whithear KG, Browning GF. A novel mechanism for control of antigenic variation in the haemagglutinin gene family of mycoplasma synoviae. Molecular microbiology. 2000;35(4):911–23. . 10692167 10.1046/j.1365-2958.2000.01766.x

[53] Buchenau I, Poumarat F, Le Grand D, Linkner H, Rosengarten R, Hewicker-Trautwein M. Expression of Mycoplasma bovis variable surface membrane proteins in the respiratory tract of calves after experimental infection with a clonal variant of Mycoplasma bovis type strain PG45. Research in veterinary science. 2010;89(2):223–9. 10.1016/j.rvsc.2010.03.014 . 20350734

[54] Dando SJ, Nitsos I, Kallapur SG, Newnham JP, Polglase GR, Pillow JJ, et al The role of the multiple banded antigen of Ureaplasma parvum in intra-amniotic infection: major virulence factor or decoy? PloS one. 2012;7(1):e29856 10.1371/journal.pone.0029856 22253806 PMC3257234

[55] Zheng X, Teng LJ, Watson HL, Glass JI, Blanchard A, Cassell GH. Small repeating units within the Ureaplasma urealyticum MB antigen gene encode serovar specificity and are associated with antigen size variation. Infection and immunity. 1995;63(3):891–8. 7868260 10.1128/iai.63.3.891-898.1995 PMC173086

[56] Robinson JW, Dando SJ, Nitsos I, Newnham J, Polglase GR, Kallapur SG, et al Ureaplasma parvum serovar 3 multiple banded antigen size variation after chronic intra-amniotic infection/colonization. PloS one. 2013;8(4):e62746 10.1371/journal.pone.0062746 23638142 PMC3637154

[57] Bertin C, Pau-Roblot C, Courtois J, Manso-Silvan L, Thiaucourt F, Tardy F, et al Characterization of free exopolysaccharides secreted by Mycoplasma mycoides subsp. mycoides. PloS one. 2013;8(7):e68373 10.1371/journal.pone.0068373 23869216 PMC3711806

[58] Klement ML, Ojemyr L, Tagscherer KE, Widmalm G, Wieslander A. A processive lipid glycosyltransferase in the small human pathogen Mycoplasma pneumoniae: involvement in host immune response. Molecular microbiology. 2007;65(6):1444–57. 10.1111/j.1365-2958.2007.05865.x . 17697098

[59] Almeida RA, Rosenbusch RF. Capsulelike surface material of Mycoplasma dispar induced by in vitro growth in culture with bovine cells is antigenically related to similar structures expressed in vivo. Infection and immunity. 1991;59(9):3119–25. 1715319 10.1128/iai.59.9.3119-3125.1991 PMC258142

[60] Tajima M, Yagihashi T, Miki Y. Capsular material of Mycoplasma gallisepticum and its possible relevance to the pathogenic process. Infection and immunity. 1982;36(2):830–3. 6177640 10.1128/iai.36.2.830-833.1982 PMC351303

[61] Maniloff J, Morowitz HJ. Cell biology of the mycoplasmas. Bacteriological reviews. 1972;36(3):263–90. 4345848 10.1128/br.36.3.263-290.1972 PMC378448

[62] Lloyd LC, Buttery SH, Hudson JR. The effect of the galactan and other antigens of Mycoplasma mycoides var. Mycoides on experimental infection with that organism in cattle. Journal of medical microbiology. 1971;4(4):425–39. . 5135333 10.1099/00222615-4-4-425

[63] Almeida RA, Wannemuehler MJ, Rosenbusch RF. Interaction of Mycoplasma dispar with bovine alveolar macrophages. Infection and immunity. 1992;60(7):2914–9. 1612758 10.1128/iai.60.7.2914-2919.1992 PMC257254

[64] Waite ER, March JB. Capsular polysaccharide conjugate vaccines against contagious bovine pleuropneumonia: Immune responses and protection in mice. Journal of comparative pathology. 2002;126(2–3):171–82. 10.1053/jcpa.2001.0540 . 11945006

[65] Garcia J, Lemercier B, Roman-Roman S, Rawadi G. A Mycoplasma fermentans-derived synthetic lipopeptide induces AP-1 and NF-kappaB activity and cytokine secretion in macrophages via the activation of mitogen-activated protein kinase pathways. The Journal of biological chemistry. 1998;273(51):34391–8. . 9852105 10.1074/jbc.273.51.34391

[66] Kacerovsky M, Celec P, Vlkova B, Skogstrand K, Hougaard DM, Cobo T, et al Amniotic fluid protein profiles of intraamniotic inflammatory response to Ureaplasma spp. and other bacteria. PloS one. 2013;8(3):e60399 10.1371/journal.pone.0060399 23555967 PMC3608618

[67] Viscardi RM. Ureaplasma species: role in neonatal morbidities and outcomes. Archives of disease in childhood Fetal and neonatal edition. 2014;99(1):F87–92. 10.1136/archdischild-2012-303351 . 23960141 PMC4239122

[68] Li YH, Brauner A, Jonsson B, van der Ploeg I, Soder O, Holst M, et al Ureaplasma urealyticum-induced production of proinflammatory cytokines by macrophages. Pediatric research. 2000;48(1):114–9. 10.1203/00006450-200007000-00020 . 10879809

[69] Li YH, Yan ZQ, Jensen JS, Tullus K, Brauner A. Activation of nuclear factor kappaB and induction of inducible nitric oxide synthase by Ureaplasma urealyticum in macrophages. Infection and immunity. 2000;68(12):7087–93. 11083834 10.1128/iai.68.12.7087-7093.2000 PMC97819

[70] Peltier MR, Freeman AJ, Mu HH, Cole BC. Characterization of the macrophage-stimulating activity from Ureaplasma urealyticum. American journal of reproductive immunology. 2007;57(3):186–92. 10.1111/j.1600-0897.2006.00460.x . 17295897

[71] Lien E, Sellati TJ, Yoshimura A, Flo TH, Rawadi G, Finberg RW, et al Toll-like receptor 2 functions as a pattern recognition receptor for diverse bacterial products. Journal of Biological Chemistry. 1999;274(47):33419–25. 10.1074/jbc.274.47.33419 . 10559223

[72] Rawadi G. Mycoplasma fermentans interaction with monocytes/macrophages: molecular basis. Microbes and Infection. 2000;2(8):955–64. 10.1016/S1286-4579(00)00395-6 . 10962279

[73] Sugiyama M, Saeki A, Hasebe A, Kamesaki R, Yoshida Y, Kitagawa Y, et al Activation of inflammasomes in dendritic cells and macrophages by Mycoplasma salivarium. Molecular oral microbiology. 2015 10.1111/omi.12117 . 26177301

[74] Xu YF, Li H, Chen W, Yao XM, Xing Y, Wang X, et al Mycoplasma hyorhinis Activates the NLRP3 Inflammasome and Promotes Migration and Invasion of Gastric Cancer Cells. PloS one. 2013;8(11). ARTN e77955 10.1371/journal.pone.0077955 . PMC3819327 24223129

[75] Sacht G, Marten A, Deiters U, Sussmuth R, Jung G, Wingender E, et al Activation of nuclear factor-kappaB in macrophages by mycoplasmal lipopeptides. European journal of immunology. 1998;28(12):4207–12. . 9862357 10.1002/(SICI)1521-4141(199812)28:12<4207::AID-IMMU4207>3.0.CO;2-R

[76] Doig PA, Ruhnke HL, Palmer NC. Experimental bovine genital ureaplasmosis. I. Granular vulvitis following vulvar inoculation. Can J Comp Med. 1980;44(3):252–8. 7427772 PMC1320070

[77] Hyatt D, Chen GL, Locascio PF, Land ML, Larimer FW, Hauser LJ. Prodigal: prokaryotic gene recognition and translation initiation site identification. BMC bioinformatics. 2010;11:119 10.1186/1471-2105-11-119 20211023 PMC2848648

[78] Galens K, Orvis J, Daugherty S, Creasy HH, Angiuoli S, White O, et al The IGS Standard Operating Procedure for Automated Prokaryotic Annotation. Standards in genomic sciences. 2011;4(2):244–51. 10.4056/sigs.1223234 21677861 PMC3111993

[79] Lowe TM, Eddy SR. tRNAscan-SE: a program for improved detection of transfer RNA genes in genomic sequence. Nucleic acids research. 1997;25(5):955–64. 9023104 10.1093/nar/25.5.955 PMC146525

[80] Bendtsen JD, Nielsen H, von Heijne G, Brunak S. Improved prediction of signal peptides: SignalP 3.0. Journal of molecular biology. 2004;340(4):783–95. 10.1016/j.jmb.2004.05.028 . 15223320

[81] Juncker AS, Willenbrock H, Von Heijne G, Brunak S, Nielsen H, Krogh A. Prediction of lipoprotein signal peptides in Gram-negative bacteria. Protein science: a publication of the Protein Society. 2003;12(8):1652–62. 10.1110/ps.0303703 12876315 PMC2323952

[82] Carver T, Thomson N, Bleasby A, Berriman M, Parkhill J. DNAPlotter: circular and linear interactive genome visualization. Bioinformatics. 2009;25(1):119–20. 10.1093/bioinformatics/btn578 18990721 PMC2612626

[83] Crabtree J, Angiuoli SV, Wortman JR, White OR. Sybil: methods and software for multiple genome comparison and visualization. Methods Mol Biol. 2007;408:93–108. 10.1007/978-1-59745-547-3_6 . 18314579

[84] Ciccarelli FD, Doerks T, von Mering C, Creevey CJ, Snel B, Bork P. Toward automatic reconstruction of a highly resolved tree of life. Science. 2006;311(5765):1283–7. 10.1126/science.1123061 . 16513982

[85] Harris JK, Kelley ST, Spiegelman GB, Pace NR. The genetic core of the universal ancestor. Genome research. 2003;13(3):407–12. 10.1101/gr.652803 12618371 PMC430263

[86] Rice P, Longden I, Bleasby A. EMBOSS: the European Molecular Biology Open Software Suite. Trends in genetics: TIG. 2000;16(6):276–7. . 10827456 10.1016/s0168-9525(00)02024-2

[87] Katoh K, Standley DM. MAFFT multiple sequence alignment software version 7: improvements in performance and usability. Molecular biology and evolution. 2013;30(4):772–80. 10.1093/molbev/mst010 23329690 PMC3603318

[88] Saitou N, Nei M. The neighbor-joining method: a new method for reconstructing phylogenetic trees. Molecular biology and evolution. 1987;4(4):406–25. . 3447015 10.1093/oxfordjournals.molbev.a040454

[89] Tamura K, Stecher G, Peterson D, Filipski A, Kumar S. MEGA6: Molecular Evolutionary Genetics Analysis version 6.0. Molecular biology and evolution. 2013;30(12):2725–9. 10.1093/molbev/mst197 24132122 PMC3840312

[90] Inoue R, Nagino T, Hoshino G, Ushida K. Nucleic acids of Enterococcus faecalis strain EC-12 are potent Toll-like receptor 7 and 9 ligands inducing interleukin-12 production from murine splenocytes and murine macrophage cell line J774.1. FEMS immunology and medical microbiology. 2011;61(1):94–102. 10.1111/j.1574-695X.2010.00752.x . 21073545

[91] Rao X, Huang X, Zhou Z, Lin X. An improvement of the 2^(-delta delta CT) method for quantitative real-time polymerase chain reaction data analysis. Biostatistics, bioinformatics and biomathematics. 2013;3(3):71–85. 25558171 PMC4280562

