# Supplementary figures and images for: Ureaplasma diversum Genome Provides New Insights about the Interaction of the Surface Molecules of This Bacterium with the Host
Source: PLoS One. 2016 Sep 7;11(9):e0161926. doi: 10.1371/journal.pone.0161926 (PMC5015763; doi:10.1371/journal.pone.0161926)

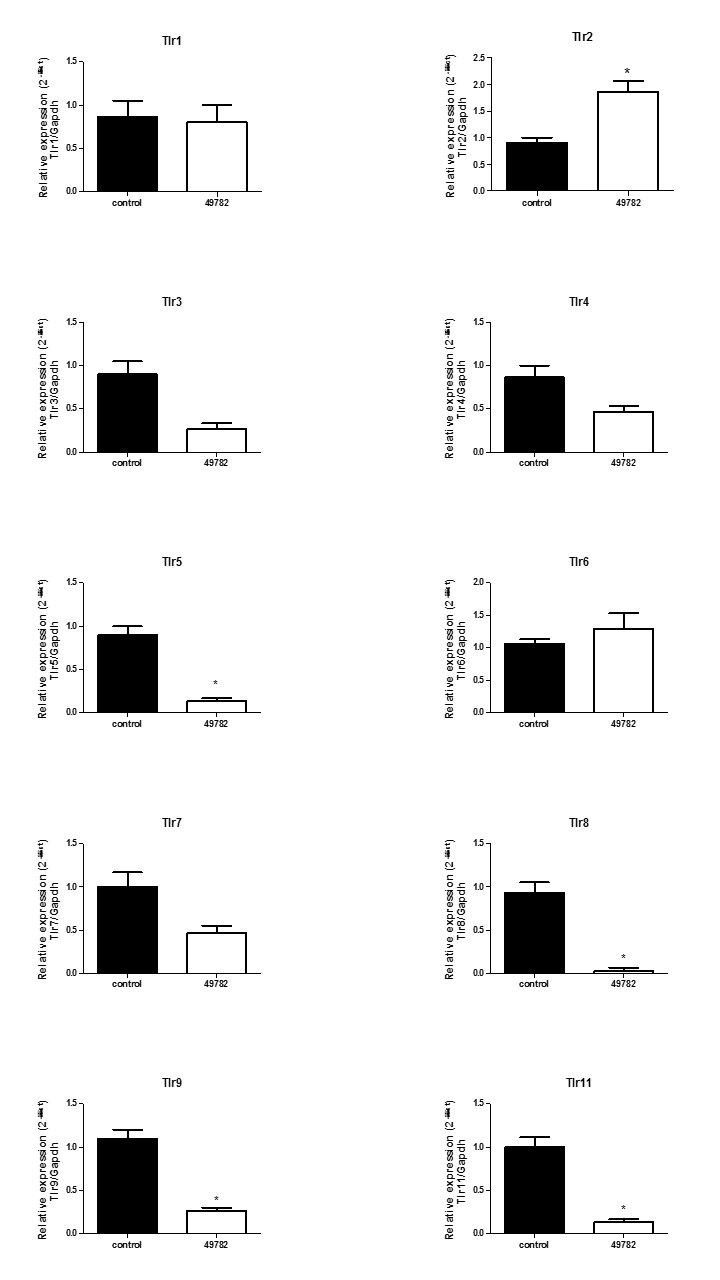

Supplement: S1 Fig — Statistical significance (p<0.05) is represented by the asterisk (*) (non-parametric Mann-Whitney analysis—One-tailed test, GraphPad Prism® version 6.01). (TIF) [file pone.0161926.s001.tif]
