# Supplementary material for: Ureaplasma diversum Genome Provides New Insights about the Interaction of the Surface Molecules of This Bacterium with the Host
Source: PLoS One. 2016 Sep 7;11(9):e0161926. doi: 10.1371/journal.pone.0161926 (PMC5015763; doi:10.1371/journal.pone.0161926)
Supplement: S1 Table — (DOCX) [file pone.0161926.s002.docx]

**Table S1**

| **Family** | **Number of CDS** | **Predicted function** | **CDS** |
| --- | --- | --- | --- |
| 1 | 16 | Hypothetical | gudiv_305hypothetical /gudiv_248conserved / gudiv_567hypothetical/ gudiv_487hypothetical / gudiv_653multiple / gudiv_618hypothetical / gudiv_505hypothetical / gudiv_304hypothetical / gudiv_391GA / gudiv_390hypothetical / gudiv_656hypothetical / gudiv_184hypothetical /gudiv_179VsaA / gudiv_137hypothetical / gudiv_657hypothetical / gudiv_182GA |
| 2 | 13 | Mobile element | gudiv_135Putative / gudiv_156integrase / gudiv_224Putative / gudiv_243Putative / gudiv_259Putative / gudiv_293Putative / gudiv_408Putative / gudiv_466Putative / gudiv_564Putative / gudiv_615Putative / gudiv_617Putative / gudiv_655Putative / gudiv_673Putative |
| 3 | 13 | Hypothetical | gudiv_136hypothetical / gudiv_155hypothetical / gudiv_225hypothetical / gudiv_244hypothetical / gudiv_258hypothetical / gudiv_292hypothetical / gudiv_409hypothetical / gudiv_465hypothetical / gudiv_565hypothetical / gudiv_614hypothetical / gudiv_616hypothetical / gudiv_654hypothetical / gudiv_672hypothetical |
| 4 | 5 | Mobile element | gudiv_436transposase/ gudiv_524transposase/ gudiv_622transposase / gudiv_623transposase / gudiv_671transposase / gudiv_795transposase |
| 5 | 5 | Hypothetical | gudiv_157Cell / gudiv_226GA / gudiv_562hypothetical / gudiv_561hypothetical / gudiv_260hypothetical |
| 6 | 5 | Mobile element | gudiv_536type / gudiv_451type / gudiv_726type / gudiv_239Type / gudiv_464type |
| 7 | 4 | Hypothetical | gudiv_633putative / gudiv_635putative / gudiv_412putative / gudiv_410putative |
| 8 | 3 | Hypothetical | gudiv_636hypothetical / gudiv_180Lipoprotein, / gudiv_177transglutaminase-like |
| 9 | 3 | Hypothetical | gudiv_506hypothetical / gudiv_490hypothetical / gudiv_438hypothetical |
