# Supplementary material for: Ureaplasma diversum Genome Provides New Insights about the Interaction of the Surface Molecules of This Bacterium with the Host
Source: PLoS One. 2016 Sep 7;11(9):e0161926. doi: 10.1371/journal.pone.0161926 (PMC5015763; doi:10.1371/journal.pone.0161926)
Supplement: S3 Table — (DOCX) [file pone.0161926.s004.docx]

**Table S3**

| **GeneBank** | **Symbol** | **Description** |
| --- | --- | --- |
| NM_001013779 | Aim2 | Absent in melanoma 2 |
| NM_009741 | Bcl2 | B-cell leukemia/lymphoma 2 |
| NM_009743 | Bcl2l1 | Bcl2-like 1 |
| NM_007465 | Birc2 | Baculoviral IAP repeat-containing 2 |
| NM_007464 | Birc3 | Baculoviral IAP repeat-containing 3 |
| NM_001163138 | Card6 | Caspase recruitment domain family, member 6 |
| NM_009807 | Casp1 | Caspase 1 |
| NM_009808 | Casp12 | Caspase 12 |
| NM_009812 | Casp8 | Caspase 8 |
| NM_011331 | Ccl12 | Chemokine (C-C motif) ligand 12 |
| NM_013653 | Ccl5 | Chemokine (C-C motif) ligand 5 |
| NM_013654 | Ccl7 | Chemokine (C-C motif) ligand 7 |
| NM_011616 | Cd40lg | CD40 ligand |
| NM_009805 | Cflar | CASP8 and FADD-like apoptosis regulator |
| NM_007700 | Chuk | Conserved helix-loop-helix ubiquitous kinase |
| NM_007575 | Ciita | Class II transactivator |
| NM_007798 | Ctsb | Cathepsin B |
| NM_008176 | Cxcl1 | Chemokine (C-X-C motif) ligand 1 |
| NM_203320 | Cxcl3 | Chemokine (C-X-C motif) ligand 3 |
| NM_010175 | Fadd | Fas (TNFRSF6)-associated via death domain |
| NM_010480 | Hsp90aa1 | Heat shock protein 90, alpha (cytosolic), class A member 1 |
| NM_011631 | Hsp90b1 | Heat shock protein 90, beta (Grp94), member 1 |
| NM_010510 | Ifnb1 | Interferon beta 1, fibroblast |
| NM_008337 | Ifng | Interferon gamma |
| NM_010546 | Ikbkb | Inhibitor of kappaB kinase beta |
| NM_010547 | Ikbkg | Inhibitor of kappaB kinase gamma |
| NM_008351 | Il12a | Interleukin 12A |
| NM_008352 | Il12b | Interleukin 12B |
| NM_008360 | Il18 | Interleukin 18 |
| NM_008361 | Il1b | Interleukin 1 beta |
| NM_133775 | Il33 | Interleukin 33 |
| NM_031168 | Il6 | Interleukin 6 |
| NM_008363 | Irak1 | Interleukin-1 receptor-associated kinase 1 |
| NM_008390 | Irf1 | Interferon regulatory factor 1 |
| NM_016849 | Irf3 | Interferon regulatory factor 3 |
| NM_013674 | Irf4 | Interferon regulatory factor 4 |
| NM_172688 | Map3k7 | Mitogen-activated protein kinase kinase kinase 7 |
| NM_011949 | Mapk1 | Mitogen-activated protein kinase 1 |
| NM_011161 | Mapk11 | Mitogen-activated protein kinase 11 |
| NM_013871 | Mapk12 | Mitogen-activated protein kinase 12 |
| NM_011950 | Mapk13 | Mitogen-activated protein kinase 13 |
| NM_011952 | Mapk3 | Mitogen-activated protein kinase 3 |
| NM_016700 | Mapk8 | Mitogen-activated protein kinase 8 |
| NM_016961 | Mapk9 | Mitogen-activated protein kinase 9 |
| NM_019453 | Mefv | Mediterranean fever |
| NM_010851 | Myd88 | Myeloid differentiation primary response gene 88 |
| NM_008670 | Naip1 | NLR family, apoptosis inhibitory protein 1 |
| NM_010870 | Naip5 | NLR family, apoptosis inhibitory protein 5 |
| NM_008689 | Nfkb1 | Nuclear factor of kappa light polypeptide gene enhancer in B-cells 1, p105 |
| NM_010907 | Nfkbia | Nuclear factor of kappa light polypeptide gene enhancer in B-cells inhibitor, alpha |
| NM_010908 | Nfkbib | Nuclear factor of kappa light polypeptide gene enhancer in B-cells inhibitor, beta |
| NM_001033367 | Nlrc4 | NLR family, CARD domain containing 4 |
| NM_001033207 | Nlrc5 | NLR family, CARD domain containing 5 |
| NM_001033431 | Nlrp12 | NLR family, pyrin domain containing 12 |
| NM_001004142 | Nlrp1a | NLR family, pyrin domain containing 1A |
| NM_145827 | Nlrp3 | NLR family, pyrin domain containing 3 |
| NM_172481 | Nlrp4b | NLR family, pyrin domain containing 4B |
| NM_001004194 | Nlrp4e | NLR family, pyrin domain containing 4E |
| NM_011860 | Nlrp5 | NLR family, pyrin domain containing 5 |
| NM_001081389 | Nlrp6 | NLR family, pyrin domain containing 6 |
| NM_194058 | Nlrp9b | NLR family, pyrin domain containing 9B |
| NM_178420 | Nlrx1 | NLR family member X1 |
| NM_172729 | Nod1 | Nucleotide-binding oligomerization domain containing 1 |
| NM_145857 | Nod2 | Nucleotide-binding oligomerization domain containing 2 |
| NM_011027 | P2rx7 | Purinergic receptor P2X, ligand-gated ion channel, 7 |
| NM_019482 | Panx1 | Pannexin 1 |
| NM_011063 | Pea15a | Phosphoprotein enriched in astrocytes 15A |
| NM_011193 | Pstpip1 | Proline-serine-threonine phosphatase-interacting protein 1 |
| NM_011198 | Ptgs2 | Prostaglandin-endoperoxide synthase 2 |
| NM_023258 | Pycard | PYD and CARD domain containing |
| NM_009045 | Rela | V-rel reticuloendotheliosis viral oncogene homolog A (avian) |
| NM_138952 | Ripk2 | Receptor (TNFRSF)-interacting serine-threonine kinase 2 |
| NM_011973 | Mok | Serine/threonine kinase 30 |
| NM_026474 | Sugt1 | SGT1, suppressor of G2 allele of SKP1 (S. cerevisiae) |
| NM_025609 | Tab1 | TGF-beta activated kinase 1/MAP3K7 binding protein 1 |
| NM_138667 | Tab2 | TGF-beta activated kinase 1/MAP3K7 binding protein 2 |
| NM_054096 | Tirap | Toll-interleukin 1 receptor (TIR) domain-containing adaptor protein |
| NM_013693 | Tnf | Tumor necrosis factor |
| NM_011613 | Tnfsf11 | Tumor necrosis factor (ligand) superfamily, member 11 |
| NM_019418 | Tnfsf14 | Tumor necrosis factor (ligand) superfamily, member 14 |
| NM_009452 | Tnfsf4 | Tumor necrosis factor (ligand) superfamily, member 4 |
| NM_009424 | Traf6 | Tnf receptor-associated factor 6 |
| NM_023719 | Txnip | Thioredoxin interacting protein |
| NM_009688 | Xiap | X-linked inhibitor of apoptosis |
| NM_007393 | Actb | Actin, beta |
| NM_009735 | B2m | Beta-2 microglobulin |
| NM_008084 | Gapdh | Glyceraldehyde-3-phosphate dehydrogenase |
| NM_010368 | Gusb | Glucuronidase, beta |
| NM_008302 | Hsp90ab1 | Heat shock protein 90 alpha (cytosolic), class B member 1 |
